# Role and implications of the CXCL12/CXCR4/CXCR7 axis in atherosclerosis: still a debate

**DOI:** 10.1080/07853890.2021.1974084

**Published:** 2021-09-08

**Authors:** Hussam A. S. Murad, Misbahuddin M. Rafeeq, Thamer M. A. Alqurashi

**Affiliations:** Department of Pharmacology, Faculty of Medicine, Rabigh, King Abdulaziz University (KAU), Jeddah, Saudi Arabia

**Keywords:** Cardiovascular, dyslipidaemia, inflammation, injury, thrombosis

## Abstract

Atherosclerosis is one of the leading causes of mortality and morbidity worldwide. Chemokines and their receptors are implicated in the pathogenesis of atherosclerosis. CXCL12 is a member of the chemokine family exerting a myriad role in atherosclerosis through its classical CXCR4 and atypical ACKR3 (CXCR7) receptors. The modulatory and regulatory functional spectrum of CXCL12/CXCR4/ACKR3 axis in atherosclerosis spans from proatherogenic, prothrombotic and proinflammatory to atheroprotective, plaque stabilizer and dyslipidemia rectifier. This diverse continuum is executed in a wide range of biological units including endothelial cells (ECs), progenitor cells, macrophages, monocytes, platelets, lymphocytes, neutrophils and vascular smooth muscle cells (VSMCs) through complex heterogeneous and homogenous coupling of CXCR4 and ACKR3 receptors, employing different downstream signalling pathways, which often cross-talk among themselves and with other signalling interactomes. Hence, a better understanding of this structural and functional heterogeneity and complex phenomenon involving CXCL12/CXCR4/ACKR3 axis in atherosclerosis would not only help in formulation of novel therapeutics, but also in elucidation of the CXCL12 ligand and its receptors, as possible diagnostic and prognostic biomarkers.

**Key messages**
1. The role of CXCL12 *per se* is proatherogenic in atherosclerosis development and progression.
2. The CXCL12 receptors, CXCR4 and ACKR3 perform both proatherogenic and athero-protective functions in various cell types
3. Due to functional heterogeneity and cross talk of CXCR4 and ACKR3 at receptor level and downstream pathways, regional boosting with specific temporal and spatial modulators of CXCL12, CXCR4 and ACKR3 need to be explored.

## Introduction

Cardiovascular diseases (CVDs) are the leading cause of death worldwide. According to the WHO, approximately 17.9 million deaths per year are caused by CVDs and this number expected to increase to more than 23.6 million deaths by 2030 [1]. CVDs comprise all heart and blood vessel malfunctions, including hypertension, coronary heart disease (CHD), coronary artery disease (CAD), acute coronary syndrome (ACS), cerebrovascular disease (stroke or transient ischaemic attack [TIA]), congestive heart failure (CHF) and peripheral vascular diseases such as aortic aneurysms, rheumatic heart disease, congenital heart diseases and cardiomyopathies. In this spectrum, CHD and CAD are the most common. Data from the American Heart Association from 2019 indicated that CHD alone killed approximately 366,000 people in 2017 in the USA [2]. CVD risk factors can include the following: age; gender; lack of physical activity; smoking; high-fat diet; alcohol consumption and other dietary and lifestyle attributes; high BMI; presence of psychosocial issues like stress, anxiety, and depression; comorbidities such as obesity, hyperlipidaemia, hypertension, diabetes and kidney diseases; family history; and genetic makeup of an individual [3,4].

## Atherosclerosis and the role of chemokines

Atherosclerosis, the most common underlying cause of CAD, is an inflammatory and fibroproliferative disease of the arterial walls with the endothelial cells (ECs), LDL molecules, platelets, leukocytes, monocytes and macrophages, intimal smooth muscle cells and chemokines playing pivotal roles. Hyperlipidaemia and shear stress initiate endothelial injury, causing the adhesion and migration of monocytes and other leukocytes, along with the permeation of lipids through the endothelium. In the subendothelial space, these lipids are oxidized and consequently become proinflammatory, chemotaxic and proatherogenic. Monocyte-derived macrophages take up these cholesterol-rich ox-LDL particles, resulting in the formation of foam cells. Endothelial dysfunction causes further migration and recruitment of other inflammatory cells through the action of several adhesion molecules and chemokines. In addition, platelets adhere to the site of the lesion and further attract more platelets and other inflammatory cells. As the lesion progresses, smooth muscle cells migrate from the media to the intima, where they proliferate; an extracellular matrix comprising elastin, collagen and proteoglycans is synthesized, leading to the creation of a fibrous cap. Meanwhile, apoptosis and necrosis of lipid-laden macrophages lead to a soft and destabilizing lipid-rich core within the plaque. In addition, cellular apoptosis contributes to thrombogenicity of the lipid-rich core. Often, neovascularization occurs and leads to plaque destabilization and damage. Superimposed thrombosis on ruptured atherosclerotic plaques is the primary cause of life-threatening clinical events [5,6].

Chemokines, which form the largest family of cytokines or cell signalling proteins, are small (8–12** **kDa) molecules that play myriad roles in various pathophysiological processes including (but not limited to) the mediation of cell mobilization; recruitment and arrest of leukocytes; activation of other cytokine members; and regulation of intracellular signalling, haematopoiesis, neurogenesis, homeostasis, autoimmune and inflammatory processes and angiogenesis [7]. Chemokines have a common basic structure; they comprise a short N-terminal region and an extended N-loop region, followed by three β-strands and one α-helix. Based on the N-terminal cysteine groups, chemokines are classified into four groups (C, CXC, CC and CX3C). Furthermore, cytokines of another group have typical chemokine activities but lack the typical chemokine structural fold and N-terminal residues; these are classified as chemokine-like factors (CLFs) [8,9].

There are two types of chemokine receptors (CKRs): conventional (cCKRs) and atypical (ACKRs). Currently, there are 18 cCKRs and four ACKRs annotated according to the predominant type of chemokine they bind. cCKRs are G-protein coupled receptors with downstream intracellular signalling, mainly including heterotrimeric G-proteins, β-arrestins and JAK‐STAT pathways. ACKRs are primarily regarded as scavenger receptors and appear to function independently of G-protein signalling pathways; nonetheless, they appear to play a role in regulating chemokine localization and function, thereby indirectly controlling interactions between chemokines and cCKRs. However, there is some controversy regarding the exact molecular mechanisms underlying the functions of ACKRs and their crosstalk with cCKRs [10]. Receptor specificity is complex, with many chemokines binding to several different CKRs; a particular CKR may have many different chemokine ligands and some non-chemokine ligands.

Chemokines and their receptors play a key role in the initiation, development, and progression of atherosclerosis. Various cells implicated in atherosclerosis pathogenesis, such as ECs, vascular smooth muscle cells (VSMCs), platelets, macrophages, monocytes and leukocytes, widely express various chemokines and their receptors [11]. Leukocyte movement through the endothelium involves several steps that are regulated by different chemokines and their receptors. Several studies have emphasized the role of different chemokines and their receptors on different stages of atherosclerosis initiation, development and progression [12]. In addition to their modulatory and regulatory functions, some chemokines directly act as adhesion molecules and some influence the phenotype of inflammatory cells. Furthermore, chemokines play a prudent role in the stability of atherosclerotic lesions, through “fibrous cap” formation. Various methodologies are being used to explore the possible roles of chemokines and their receptors in atherosclerosis, including genetic and pharmacological experiments. For example, atherosclerosis was induced in ApoE−^/^− or Ldlr−^/^− knockout murine models through vascular injury or a high-fat diet; furthermore, a specific chemokine or receptor was knocked out to ascertain its role in atherosclerosis [13].

Signalling of the chemokine CXCL12 (stromal cell-derived factor 1 [SDF-1]) through its receptors CXCR4 and ACKR3 (CXCR7) forms the CXCL12/CXCR4/ACKR3 axis or the CXCL12/CXCR4/ACKR3 pathway. The importance of this axis is increasingly being acknowledged in various pathologies such as CVDs, autoimmune diseases, homeostasis, cardiac and neural development and cancers. However, this review focuses specifically on the role of CXCR4 and CXCR7 (ACKR3) receptors, their complex mutual relationship and their interaction with the ligand CXCL12 (SDF-1) in atherosclerosis. To gain a greater understanding of the role played by the CXCL12/CXCR4/ACKR3 axis in atherosclerosis and other pathologies, it is imperative to create cell-specific knockout models as well as conditional and inducible knockout models of the ligand and the receptors. Many studies have been conducted on different chemokines, their receptors, and their role in CVDs, but there is still a large knowledge gap in understanding the exact underlying mechanisms.

## CXCL12/CXCR4/ACKR3 axis

### CXCL12

CXCL12, also known as SDF-1, is secreted by the stromal cells from the basolateral side. On the endothelium, it interacts with glycosaminoglycans, as is required for its stability and presentation to leukocytes. CXCL12 is a member of the CXC chemokine family, which consists of 17 members and can be further categorized into two groups based on the presence of the [glutamate–leucine–arginine (ELR)] amino-acid motif preceding the first cysteine group. The ELR − chemokine subgroup, which also includes CXCL12, mainly attracts NK cells and T-lymphocytes, while the ELR + subgroup attracts neutrophils [14,15]. There are six different isoforms of CXCL12, viz. CXCL12-α, -β, -γ, -δ, -ε and –φ, based on alternative splicing of the CXCL12 gene. The N-terminal sequence is the same in all isoforms, while the C-terminal region varies. The α and β isoforms are expressed ubiquitously, while the rest have a limited expression pattern [16]. CXCL12 plays a prominent role in haematopoiesis, angiogenesis, immunogenesis, stem cell mobilization and neurogenesis, as evidenced by a defect in these processes in CXCL12-knockout mice [17]. These findings were further reinforced by decreasing CXCL12 levels through its receptor CXCR4 antagonist AMD3100, resulting in reduced mobilization of stem cells from the bone marrow [18].

CXCL12 is also expressed in the form of monomers or dimers as mediated by interaction with GAG, and these forms remain in equilibrium and are regulated by cellular pH. Monomers are reported to bind preferentially to atypical receptors, while both forms display differences while interacting with classical receptors, resulting in myriad variations in downstream signalling [19,20]. Early studies on its role in the vascular system have indicated that CXCL12 accelerates the healing process in vascular injury and supports ischaemic neovascularization. This is evident from the enhanced endothelial progenitor cell (EPC) recruitment in ischaemic tissues after CXCL12 injections [21]. In another vascular injury model, CXCL12 increased the formation of carotid neointima through the recruitment of smooth muscle cells [22]. The detailed description of CXCL12 in different pathophysiological processes is beyond the scope of this review. Nonetheless, its role in atherosclerosis has been discussed elsewhere.

### CXCR4

CXCR4 is the main specific receptor for CXCL12 and serves as an amplifier to increase CXCL12-associated signalling. It is widely expressed on various cell types that play a role in CVDs, including ECs, VSMCs, T-cells, B-cells, neutrophils, monocytes, and macrophages. CXCR4 is a 7-α helical transmembrane G protein-coupled receptor, and its binding with its ligand, CXCL12, induces intracellular signalling *via* a classical heterotrimeric G protein [23]. The different G proteins activated by CXCR4 include Gα_i1_, Gα_i2_, Gα_i3,_ Gα_q_ and G_αo_, with maximum efficiency reported for the Gα_i1_ and Gα_i2_ subtypes [24]. In addition, other non-cognate G proteins may be activated in specific contexts, such as metastatic breast cancer. Receptor activation leads to multiple downstream signalling pathways, including activation of the mitogen-activated protein kinase/extracellular signal-regulated kinases (MEK/ERK) pathway, phospholipase C (PLC) beta and gamma2, phosphoinositide 3-kinase/protein kinase B (PI3K/Akt/mTOR) pathway and nuclear factor κB (NF-κB) signalling through G protein; other pathways like Ca^2+^/calmodulin dependent protein kinase II/AMP-response element-binding protein (CaMKII/CREB), Wnt/β-catenin and Janus kinase/signal transducer and activator of transcription protein (JAK/STAT) pathways can also be activated independent of G protein [25–29]. All these pathways are involved in the pathogenesis of vasculopathy, local inflammation, chemotaxis and proliferation [30–32]. Receptor internalization and degradation are mediated by the phosphorylation of specific kinases through the recruitment of β-arrestins. Different cellular locations and microenvironments also play a role in CXCR4 signalling. For example, CXCR4 localization to cholesterol-containing membrane rafts plays a role in CXCL12 binding, while in metastatic renal carcinoma, CXCR4 is localized to the nucleus. Hence, potential therapeutic targeting of CXCR4 warrants further information on the precise location and expression of CXCR4.

CXCR4 is also a receptor for macrophage migration inhibition factor (MIF), which is a Chemokine Like Factor (CLF), and it is implicated in various pathological processes such as chemotaxis, leukocyte recruitment, inflammation and epithelial-mesenchymal interaction in tumours. MIF also acts as a partial allosteric agonist of CXCR4 [33,34]. CXCR4 heterodimerizes with other receptors, such as CXCR3, the Na+/H + exchanger regulatory factor 1 (NHERF1), CCR7, CCR2, CCR5, α1-AR and opioid receptors, resulting in differential signalling; however, their roles in atherosclerosis are still uncertain [35]. CXCR4/CXCR3 heterodimerization delays CXCR4 signalling [36]. CXCR4 itself is expressed in monomeric or dimeric forms, and they are both located at the outer cell membrane and intracellular structures [37]. Recently, it has been reported that actin-dependent nanoclustering of CXCR4 is also required for optimal CXCL12 signalling [38,39]. Complex interactions of CXCR4 with another receptor, CXCR7 (ACKR3), are discussed below.

### ACKR3 (CXCR7)

ACKRs are structurally similar to classical chemokine G-protein coupled receptors, but because of the absence of a specific amino acid motif, they do not couple with G-proteins. Hence, there is no induction of G-protein-dependent intracellular signalling pathways necessary for chemotaxis and leukocyte recruitment. However, ACKRs are implicated in the internalization, scavenging, and regulation of chemokine bioavailability [40]. They are expressed on red blood cells, neurons, ECs, some leukocyte subsets, platelets, arteriole smooth muscle cells, monocytes and mature B cells. Besides, its role in the heart and blood vessels, ACKR3 has a prudent role in the development of the cardiovascular system. This is evident from the presence of cardiac valvular defects in ACKR3-knockout mice, resulting in perinatal mortality [41,42].

ACKR3 has 10 times more affinity for CXCL12 than the classical receptor CXCR4 and is generally considered a negative regulator of CXCL12 expression and function. Specific ACKR3 inhibitors such as CCX771 have been shown to increase the plasma levels of CXCL12 by several folds [43]. In addition to CXCL12, ACKR3 has other ligands such as CXCL11, MIF (non-cognate), adrenomedullin (ADM) and the viral chemokine vCCL2/viral macrophage inflammatory protein-II [30,44]. The primary role of ACKR3, as documented initially, is to internalize and deliver its ligand CXCL12 for lysosomal degradation, thus regulating the CXCL12/CXCR4 signalling cascade. Continuous cycling between lysosomes and the plasma membrane establishes a gradient for CXCL12 [45]. Conversely, the phosphorylation and subsequent prevention of degradation of ACKR3 by CXCL12 are responsible for the regulation of equilibrium between membrane-bound and intracellular ACKR3.

ACKR3 has also been reported to be involved in signalling, independent of G-protein, through β-arrestin-2 (even with greater strength than CXCR4), which is implicated in signalling and desensitization. In addition, β-arrestin-2 signalling has been reported to play a role in cell survival and growth through the activation of Akt [46]. This hypothesis was reinforced by depleting cellular β-arrestin-2 and cleaving the ACKR3 carboxyl-terminus, thus attenuating ACKR3 signalling [47]. In addition, the binding of another ligand MIF to ACKR3 mediates other pathways such as phosphoinositide-3-kinase-Akt signalling, JAK2/STAT3 pathway and ERK1/2 pathway [48,49].

Further complex interactions have evolved through heterodimerization with CXCR4 [50]. ACKR3 is co-expressed with CXCR4 in a wide variety of cells, such as T and B cells, dendritic cells, monocytes, VSMCs and ECs [51,52]. The interaction between ACKR3 and CXCL12/CXCR4 is complex. For example, on one hand, CXCL12 degradation by ACKR3 scavenging results in the inhibition of CXCR4 downregulation, but on the other hand, ACKR3 stimulation directly results in CXCR4 downregulation [50,53]. Although overexpression of ACKR3 has been reported to attenuate CXCR4-mediated Ca^2+^ response, whether this phenomenon also applies to endogenous ACKR3 is not clear [54]. Moreover, another study reported an increase in CXCR4-mediated Ca^2+^ response after the depletion of endogenous ACKR3 by RNA interference [55].

Conversely, CXCR4 also seems to influence ACKR3, as the CXCR4 antagonist AMD3100 has shown agonistic activity against ACKR3 [56]. In addition, the heterodimerization of CXCR4/ACKR3 seems to interfere with CXCR4-induced Gαi protein-mediated signalling but activates signalling pathways such as ERK1/2, p38/mitogen-activated protein kinase (MAPK), and ste20-related proline/alanine-rich kinase (SPAK) through β-arrestin-2 [57]. A previous study suggested that there may be a physical interaction between the two receptors on finding that ACKR3 inhibition negatively regulates CXCR4-mediated lymphocyte integrin adhesiveness in T-cells [58]. It has also been reported that CXCL12, while mediating CXCR4 internalization and degradation, favours the externalization of ACKR3 from the membrane of endocytic vesicles to the plasma membrane in platelets. This is regulated by CXCR4 and was confirmed in the absence of ACKR3 externalization, post CXCR4 blockade [59].

It has not yet been fully elucidated whether CXCR4/ACKR3 heterodimerization, CXCR4 homodimerization, or the equilibrium ratio of CXCL12 monomers/dimers is more crucial for preferential receptor signalling. This is because of the lack of experimental data due to the non-availability/non-efficacy of synthetic peptides that prevent heteromerization. It is also speculated that in addition to the physical heterodimerization of CXCR4/ACKR3, the convergence of downstream signalling pathways or concurrent activation of parallel signalling pathways can lead to CXCL12 differential responses in different cells, as evidenced by increased recruitment and potentiation of ß-arrestin-2-dependent cell signalling pathways [57]. With respect to ß-arrestin-2, a combined experimental and computational approach revealed an interesting result: CXCL12 induced a rapid short-term increase in the interaction between ß-arrestin-2 and CXCR4as well as a prolonged steady interaction between ß-arrestin-2 and ACKR3 in cells expressing either of these receptors. However, in cells co-expressing CXCR4 and ACKR3, there was a marked attenuation in the CXCL12-mediated interaction between ß-arrestin-2 and CXCR4, without a significant effect on the interaction between ß-arrestin-2 and ACKR3. Hence, ß-arrestin-2 appears to play a crucial role in CXCL12-mediated CXCR4/ACKR3 integrated responses [60].

Furthermore, in some cells, there is a complete loss of CXCL12 signalling after the blockade of either CXCR4 or ACKR3, while in other cells, there is only a partial loss; this indicates the presence of both synergistic and additive actions depending on the cellular environment. However, it is also hypothesized that the additive effect might be owing to receptor subpopulations. In addition, CXCL12 signalling is modulated by not only mutual CXCR4/ACKR3 homo- or hetero-dimerization, but also complex interactions with other non-CXCL12 receptors, non- CKRs, and non-receptor proteins [61]. Hence, from the above discussion, it is clear that the crosstalk between CXCR4 and ACKR3 is complex and warrants further sophisticated experimentation.

## CXCL12/CXR4/ACKR3 axis in atherosclerosis

CXCL12 is considered proatherogenic because of its involvement in dyslipidaemia, angiogenesis, plaque destabilization, thrombus formation, neointimal hyperplasia, and proinflammatory effects on the vascular endothelium [62]. However, external administration of CXCL12 in ApoE−/− mice models has been shown to promote stabilization of atherosclerotic lesions through the accumulation of smooth muscle cells, increase in collagen content, and thickening of the fibrous cap, without altering the vessel lumen diameter[63]. Nevertheless, it should be noted that ligated artery lesions in the above report were phenotypically different from lesions in normal vessels.

Significantly greater mRNA expression of CXCR4, ACKR3 and CXCL12 was found in patients with carotid plaques who reported to cardiovascular clinics [64]. In addition, an increase in CXCR4-positive cells has been reported with progression of atherosclerotic plaques [65]. The CXCL12/CXCR4/ACKR3 axis performs distinct functions in the pathophysiology of atherosclerosis, ranging from atheroprotective to proatherogenic functions across various cell types, through its receptors CXCR4 and ACKR3 (Table 1). In addition, cross-talk of CXCR4/ACKR3 together with their interaction with ligands other than CXCL12 further increases the intricacy of these functions [83]. The cell-specific and process-specific atheroprotective and proatherogenic functions of the CXCL12/CXR4/ACKR3 axis are discussed below.

**Table 1.** Showing the predominant effect of CXCL12/CXCR4/ACKR3 axis in different cells involved in atherosclerosis.

	Receptor	Cell type	Effect	References
**C** **X** **C** **L** **1** **2**	ACKR3	Macrophages	Aggravation	[66,67]
	ACKR3	Leukocyte recruitment	Aggravation	[38]
	ACKR3	Platelet	Protection	[68]
	ACKR3	Lipids	Protection	[69]
	ACKR3	Vascular smooth muscle cell	Protection	[68]
	CXCR4/ACKR3	Platelet	Aggravation	[49]
	CXCR4	Endothelial cell (eNOS)	Aggravation	[70,71]
	CXCR4	Neutrophil	Protection	[72]
	CXCR4/ACKR3	Endothelial progenitor cell (migration & proliferation)	Protection	[73–75]
	CXCR4	Vascular smooth muscle cell	Protection/aggravation	[76]
	CXCR4	Cardiomyocyte	Aggravation	[77]
	CXCR4	Macrophage	Aggravation	[78]
	CXCR4	Dendritic cell	Aggravation	[79]
	CXCR4/ACKR3	Endothelial progenitor cell (adhesion)	Aggravation	[66]
	CXCR4/ACKR3	Monocytes	Aggravation	[49]
	CXCR4	Endothelial cells	Protection	[80].
	ACKR3	Endothelial cells	Protection	[81]
	CXCR4	B-cells	Protection	[82]

### Endothelial injury and repair

EPCs, smooth muscle progenitor cells and vascular ECs are key regulators of repair mechanisms after vascular insult. After vascular endothelial injury, EPCs enhance vascular repair, promote vascular integrity and reduce neointima and atherosclerotic plaque formation. However, EPCs are also involved in neo-angiogenesis, leading to plaque instability and thrombus formation. The CXCL12/CXCR4/ACKR3 axis plays a pivotal role in the migration, proliferation, survival and adhesion of EPCs. Tissue hypoxia also upregulates CXCL12 and CXCR4 [84,85].

CXCR4 and ACKR3 are strongly expressed in ECs and EPCs. CXCL12-induced recruitment and migration of EPCs are predominantly regulated by CXCR4, while adhesion and survival of EPCs are mainly regulated by ACKR3; hence, both are essential for endothelial repair and re-endothelialization. However, it is also speculated that ACKR3-mediated EPC adhesion may eventually lead to trans-endothelial leukocyte adhesion and migration, contributing to atherosclerosis [75,86].

It is noteworthy that ACKR3 also plays a positive role in plaque stabilization and fibrous-cap formation through VSMC recruitment and migration (albeit through CXCL11) [68]. Recently, CXCR4 has also been reported to be atheroprotective with reference to VSMCs, as VSMC-specific CXCR4 deficiency in mice led to increased lesion progression. CXCR4 deficiency also switches VSMCs from a contractile to a secretory phenotype (macrophage-like), favouring atherosclerosis. However, bone marrow-derived VSMCs play a role in aggravating atherosclerosis through neointimal hyperplasia.

Chen et al. [73] demonstrated that CXCL12 protects the endothelium by reducing endothelial permeability and enhancing endothelial integrity through the CXCR4/PI3K/Rac1 signalling pathway (but not through ACKR3). They found an attenuated response to CXCL12-induced endothelial barrier enhancement, along with increased endothelial permeability after CXCR4 blockade, but not after ACKR3 blockade [73,74]. In contrast, another study demonstrated a protective role of ACKR3, albeit in normolipidemic myocardial infarction and endothelial denudation injury mouse models, and reported that the loss of endothelial ACKR3 increased neointimal hyperplasia [81].

The CXCR4-mediated endothelial protection theory was further reinforced by [80], who demonstrated reduced endothelial regeneration and increased neointimal hyperplasia in an EC CXCR4-knockout mouse model [80]. They also reported enhanced healing after stimulation of human aortic ECs by CXCL12, which was blunted by the CXCR4 antagonist, AMD3100. Furthermore, Döring et al. demonstrated that CXCR4 maintains vascular integrity through the WNT/β-catenin pathway, VE-cadherin expression and function and stabilization of junctions [87]. It is also interesting to note that during atherosclerosis, EC-derived apoptotic bodies are formed, which trigger the production of CXCL12 through microRNA-126, consequently trigger the recruitment of progenitor cells, and lead to plaque stability [88].

Hence, it is clear from the above discussion that CXCR4 is prudent in maintaining endothelial integrity and reducing neointimal formation. However, CXCR4-mediated recruitment of other types of progenitor cells that later differentiate into smooth muscle-like cells and the presence of CXCR4 on VSMCs and leukocyte subsets may contribute to neointimal formation [89]. In addition, some proatherogenic effects of CXCR4 have been demonstrated in CXCR4-carrying haematopoietic cells and other bone marrow-derived cells, but the exact significance and underlying mechanism are not well understood [87].

For a clearer picture of the association between EC CXCL12/CXCR4 and atherosclerosis, five different models of CXCL12-knockout were created, and the lesion area, macrophage content and collagen content were studied. The lesions improved in EC-knockout models specifically, along with a decrease in the level of circulating CXCL12, suggesting that EC-derived CXCL12 promotes atherosclerosis. However, in contrast to EC CXCL12 knockout, EC CXCR4 knockout caused endothelial leakage and leukocyte invasion, implying that EC CXCR4 protects against atherosclerosis by preserving endothelial integrity and barrier function [17].

CXCL12/CXCR4 binding also increases the induction of eNOS phosphorylation through the PI3K/Akt pathway [71]. Under normal physiological conditions, eNOS protects against atherosclerosis, but it leads to angiogenesis and plaque destabilization in atherosclerotic vascular injury [70]. Hence, as angiogenesis progresses through EC and EPC recruitment, stimulated by CXCL12, there is a chance of increased plaque vulnerability and thrombus formation [90].

### Dyslipidaemia and inflammation

CXCL12 levels are also associated with hyperlipidaemia. Serum CXCL12 was found to be elevated in hyperlipidaemia patients, suggesting its role as a biomarker [91]. In addition, statins, which are used to treat hyperlipidaemia, cause a decrease in CXCL12 levels. It is reported that hypercholesterolaemia disturbs the bone-marrow CXCL12/CXCR4 axis, generating a proatherogenic state [92]. Furthermore, CXCL12 expression was found to be upregulated with increased LDL levels and inversely related to HDL levels [93]. CXCL12 overexpression in ApoE−/− mice demonstrated increased macrophage infiltration, reduced functioning of reverse cholesterol transport, decreased plasma HDL-C levels and enlargement of atherosclerotic lesions. Moreover, the CXCL12/CXCR4 interaction activates the GSK-3β/β-catenin^T120^/TCF21 signalling pathway to inhibit ABCA1-dependent cholesterol efflux from macrophages to Apo-A1 and aggravate atherosclerosis [84,85].

Additionally, ACKR3 is considered atheroprotective as it reduces hypercholesterolaemia. A reduction in adipose tissue lipid uptake after ACKR3 knockout in a carotid injury atherosclerotic model was demonstrated. The ACKR3 ligand (CCX771) also reduced lipid levels and monocyte-induced macrophage formation in ApoE−/− mice, thus reducing atherosclerotic lesions [69]. Moreover, atorvastatin which is used to lower cholesterol, decreases the mRNA and protein expression of ACKR3 in macrophages, leading to attenuated macrophage migration [86]. Recently, ACKR3 was reported to limit atherosclerosis initiation by modulating the pyroptosis pathway, which is a highly inflammatory lytic form of apoptosis. ox-LDL-induced pyroptotic cell death was ameliorated by ACKR3 (activated by its agonist TC14012) in ApoE−/− mice fed a high-fat diet [94].

However, in contrast to the above observations, higher concentrations of ACKR3 were found in the macrophages of aortic atherosclerotic lesions in ApoE−/− mice than in healthy tissues [49] suggested that ACKR3 plays a role in monocyte function during inflammation by demonstrating increased CXCR4, ACKR3 and CXCL12 expression. They further demonstrated that ACKR3 expression on monocytes is crucial for monocyte survival and adhesion to platelet-secreting CXCL12 [49]. During the process of monocyte-induced macrophage formation in the intima, ACKR3 induction and expression occurs, which changes the CXCL12 signalling downstream pathways from pro-survival (ERK, Akt) to pro-inflammatory (JNK, p38) in macrophages and leads to increased phagocytosis and atherogenesis [66]. It has also been reported that CXCL12 is upregulated, which boosts monocyte chemoattractant protein-1 and leads to adhesion of monocytes to ECs. In addition to ACKR3, CXCR4 enhances ox-LDL uptake and foam cell formation and promotes atherosclerosis. ox-LDL upregulates CXCR4 expression and increases CXCL12 release from macrophages, thereby increasing foam-cell formation. In addition, blockage of CXCR4 has been shown to reduce macrophage content in neointimal lesions [95].

The CXCL12/CXCR4 axis regulates neutrophil haemostasis. CXCR4 is highly expressed in senescent neutrophils. There is preferential trafficking of senescent neutrophils to bone marrow through CXCR4‐dependent manner. Binding of CXCL12 or other agonists to neutrophil CXCR4 induces TNF-related apoptosis-inducing ligand (TRAIL) and TRAIL death receptor expression leading to an accelerated apoptosis of senescent neutrophils in the bone marrow. It has been demonstrated that disruption of neutrophil senescence, apoptosis, and systematic removal by blocking CXCR4 leads to aggravation of atherosclerosis, as there is increased adhesion and migration [72]. Moreover, blockade of bone-marrow CXCR4 by AMD3465 leads to increased neutrophil content in atherosclerotic lesions. CXCL12 also plays a role in the maturation of dendritic cells, which then regulate T cells. T cells secrete IFN-γ, leading to further inflammation and endothelial T-cell infiltration and aggravating the lesion and plaque [79]. In addition, CXCR4 plays an important role in B-cell and T-cell migration and chemotaxis, albeit through MIF [96]. Recently, the role of CXCR4-expressing B-1 cells has been explored. Doring et al. demonstrated that in female B-cell-specific Cxcr4-deficient ApoE−/− Western diet fed mice, bone-marrow B-1 cells and IgM reduced significantly without any change in CXCL12. Moreover, there was a significant increase in the size of atherosclerotic lesions along with an increase in plaque macrophage content [82]. Upadhye et al. also demonstrated a protective role of B-1 cell-mediated IgM secretion in plaque progression and mentioned that CXCR4 expression is a critical factor that plays a role in bone-marrow B-1a cell localization and IgM production [97]. Hence, this shows the protective role of B-1 cell-specific-CXCR4 in atherosclerosis

### Platelets

Platelets arrive at the site of vascular injury and contribute to endothelial activation through their glycoproteins. They express CXCR4 receptors and produce CXCL12, which plays a crucial role in cell adhesion and chemotaxis. Moreover, CXCL12 induces platelet aggregation and transmigration [98]. Platelet-derived CXCL12 appears to act in a paracrine manner through CXCR4 signalling by increasing dense granule secretion and thromboxane A2 production and consequently leading to thrombus formation, as a CXCR4 blockade significantly attenuated these functions [99].

Increased platelet survival and reduced apoptosis by ACKR3 (through the MIF ligand) also help to stabilize plaques [68]. Circulating CXCL12 enhances ACKR3 expression and decreases CXCR4 expression on platelets, leading to increased platelet survival and plaque stabilization. However, platelet-released CXCL12 mediates platelet aggregation as well as increases foam-cell formation and monocyte migration (through CXCR4), adhesion, survival (through ACKR3) and their differentiation into CD163^±^ macrophages possibly through CXCR4/ACKR3 bidirectional trafficking, making the lesion more atherogenic. Similarly, the CXCL12/CXR4 interaction in VSMCs promotes plaque stabilization through cross-talk with EGFR, but aggravates the lesion by increasing intimal hyperplasia (in a restenosis model) and promoting leukocyte recruitment [76]. Likewise, CXCL12 facilitates the removal of apoptotic platelets by monocytes and macrophages, but enhances the conversion of macrophages to foam cells, thus promoting atherosclerosis [49,95].

Clinically, surface expression of both ACKR3 and CXCR4 was significantly enhanced in platelets in ACS patients compared to that in subjects with stable CAD [100]. This finding was supported by another study reporting that CXCL12 upregulates ACKR3 surface availability on platelets [59]. It has also been reported that decreased platelet CXCR4 expression in CAD patients is associated with an increase in cardiovascular events and all-cause mortality, while increased ACKR3 is associated with improved outcomes [101].

**Figure 1.**
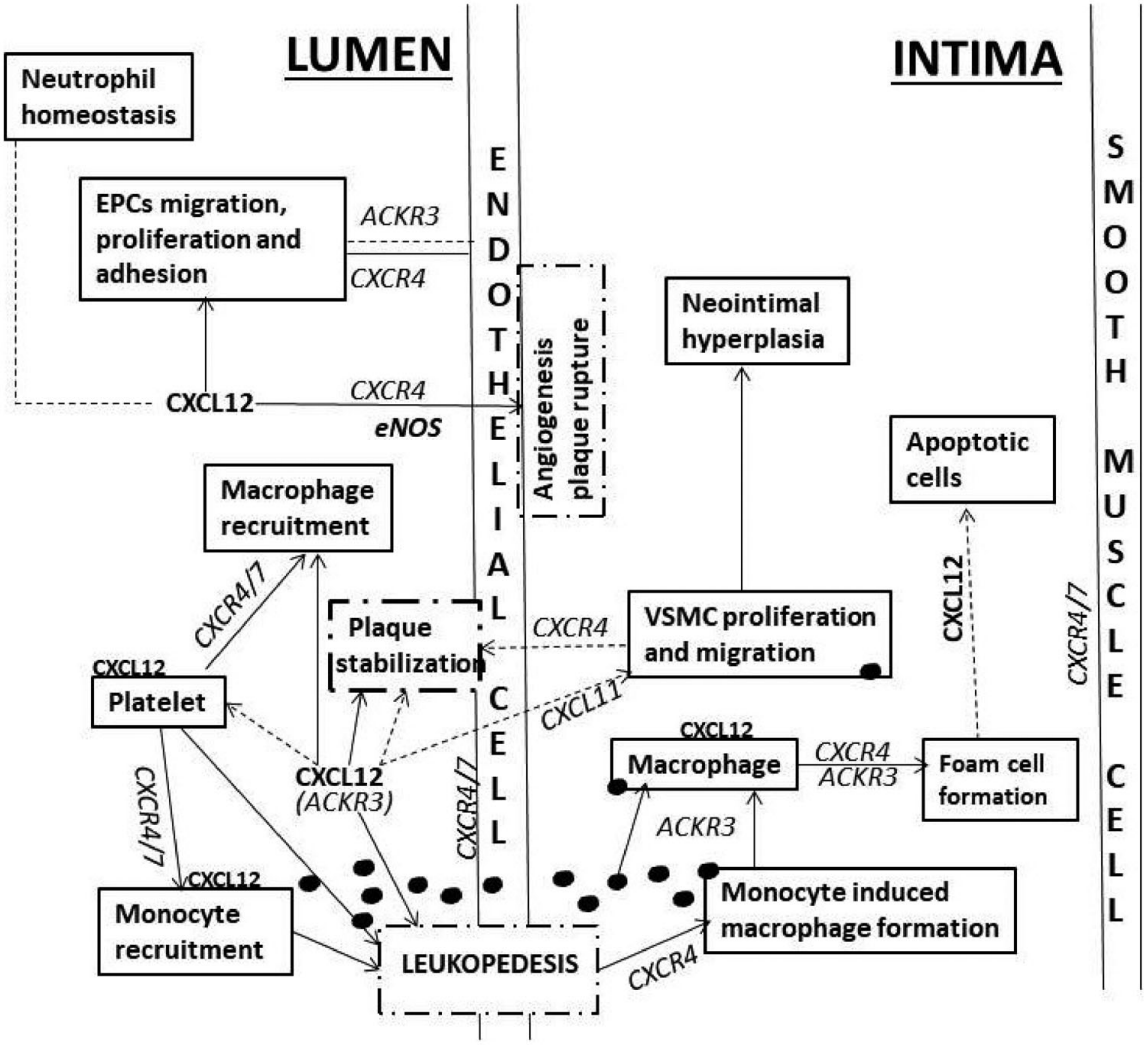
Figure 1 showing the possible role of CXCL12/CXCR4/ACKR3 axis in the pathogenesis of atherosclerosis. Black dots: oxLDL; EPCs: endothelial progenitor cells; VSMC: vascular smooth muscle cell. The black dot attached with box denotes lipid intake. Letters in italics are receptors; letters in bold are chemokines; arrows show proatherogenic role; dotted arrows show atheroprotective role. Large font CXCL12 denotes circulating CXCL12; small font CXCL12 denotes CXCL12 secreted from various cells. CXCR4/7 shows dimerization of two receptors or ambiguous role. Circulating CXCL12 is proatherogenic per se. But it also enhances the migration and proliferation of EPCs and EC repair through CXCR4, and adhesion through CXCR7. CXCL12/CXCR4 also increases the induction of eNOS phosphorylation leading to angiogenesis and plaque destabilization. CXCL12 also exerts a protective role by maintaining neutrophil homeostasis through CXCR4. Platelet-derived CXCL12 mediates platelet aggregation, increased foam cell formation, monocyte migration and adhesion through CXCR4/ACKR3 (depicted as CXCR4/7). But circulating CXCL12 enhances ACKR3 expression on platelets leading to increased survival of platelet and plaque stabilization and an atheroprotective action. ACKR3 and CXCR4 are also involved in plaque stabilization and fibrous cap formation through VSMC migration and proliferation. Both ACKR3 and CXCR4 are involved in uptake of ox-LDL and monocyte to macrophage induction and foam cell formation, which further attract inflammatory cells recruitment leading to atherogenic lipid core. Ox-LDL also aggravates CXCL12 release from macrophages. Detailed processes are not shown.

Put together, as discussed above in detail and summarized in Figure 1, the CXCL12/CXCR4/ACKR3 axis plays dual roles of both athero-protection and athero-aggravation. CXCL12 is predominantly proatherogenic, while the receptors CXCR4 and ACKR3 have cell-specific and process-specific functional outcomes.

## Clinical implications

Since the CXCL12 gene was first identified by a genome-wide association study (GWAS) as a novel locus with a significant role in CVDs [102], various other GWASs have shown a strong link between the CXCL12 gene and CVDs [103]. For example, rs1746048 at locus 10q11 is associated with higher plasma CXCL12 levels and increased risk for CAD with age and gender adjustments. Similar findings were reported in another study using the same single nucleotide polymorphism (SNP) [104,105]. Likewise, an SNP in the CXCL12 gene (rs1801157) was found to be associated with CHD in Chinese patients [106]. Another study associated the prognostic value of several SNPs in the CXCL12 gene with outcomes of CAD in a predominantly Caucasian population [107]. Recently Sjaarda et al. advocated a causal relationship between elevated CXCL12 levels and CAD through Mendelian randomization analysis of several biomarkers from two large consortia [108].

It was also reported that atherosclerotic patients with higher CXCL12 levels are at an increased risk for developing thrombosis. Furthermore, increased platelet expression of CXCL12 in CAD patients was found to be associated with adverse clinical outcomes (Dominik [109]. Because platelet-derived CXCL12 is expressed very early in ACS, it can be evaluated as a potential early biomarker [110]. In addition, as mentioned elsewhere, increased serum CXCL12 levels are found in patients with dyslipidaemia, and statins used for the treatment of dyslipidaemia have been shown to reduce CXCL12 levels (probably through ACKR3), implicating the CXCL12/ACKR3 axis as a potential therapeutic target. CXCL12 levels also have an inverse relationship with HDL-C levels, but the exact role of CXCL12 in regulating dyslipidaemia is still unclear [111].

Except for a few clinical reports advocating the protective role of CXCL12 on the basis of the clinical finding of decreased serum CXCL12 levels in stable and unstable angina [112], almost all studies emphasize a proatherogenic role of CXCL12. Hence, in addition to evidence from the genetic studies mentioned above, CXCL12 has been associated with atherogenic dyslipidaemia, pro-thrombotic activity, poorer cardiovascular outcomes and progression of atherosclerosis (after endothelial injury), making it a potential biomarker and therapeutic target.

CXCR4 is also being considered as a surrogate biomarker for atherosclerotic plaque development and progression owing to its differential expression and function in macrophages, ECs, EPCs, VSMCs, platelets, and atherosclerotic plaques, in addition to its role in neutrophil homeostasis and bone marrow-derived cell migration and proliferation. It has also been hypothesized that acute blockade of CXCR4 by its antagonist AMD3100 improves cardiac outcome [113] while long-term suppression of CXCR4 exacerbates cardiac dysfunction [114]. Moreover, results from genetic data mining and molecular radio-imaging studies discussed below also support this notion.

Recently, a search for the identification of genes and pathways involved in atherosclerotic plaque progression and rupture was performed using bioinformatics analysis of relevant human genome datasets from NCBI. Of the 29 common differentially expressed and upregulated genes in early and advanced plaques (the GSE28829 dataset) and in stable and ruptured plaques (the GSE41571 dataset), expression of the CXCL12 and CXCR4 genes was markedly enriched [115]. This finding was further confirmed by qRT-PCR in aortic samples of a murine atherosclerotic model, which also revealed significantly increased mRNA expression of CXCR4 and CXCL12 genes (R. [115]. Earlier, Doring (2017) found that patients with the homozygous C allele at SNP rs2322864 had lower CXCR4 mRNA expression in atherosclerotic plaques [87]. In addition, CXCR4 expression was reduced in symptomatic patients than in asymptomatic patients, suggesting the role of vascular CXCR4 as a therapeutic target.

Hyafil et al. reported the increased expression of CXCR4 and accumulation of CXCR4-positive macrophages in tissue samples of human carotid plaques through radionuclide ^68^Ga-pentixafor PET imaging [116]. These findings were further reinforced by another study [117]. Likewise, ^68^Ga-pentixafor, which is a CXCR4 ligand for PET, had an increased uptake in vulnerable plaques and in plaques of patients with atherosclerotic risk factors such as dyslipidaemia, smoking, hypertension and previous cardiovascular events [118]. Furthermore, Li et al. performed CXCR4-targeted ^68^Ga-pentixafor PET imaging of human carotid arteries for *in vivo* quantification of CXCR4 and found that ^68^Ga-pentixafor uptake was significantly higher in moderate to severe eccentric carotids than in non-eccentric carotids. Moreover, histochemical assessment of carotid stenosis patients revealed that enhanced CXCR4 expression is localized in inflamed atherosclerotic plaques [119].

Another molecular radio-imaging study employing a CXCR4-specific tracer in a murine model of atherosclerosis revealed increased EC CXCR4 expression in sites of endothelial injury and progressive plaques, which were correlated with increased monocyte recruitment, implying that CXCR4-targeted molecular imaging might be utilized in monitoring plaque injury, plaque progression and monocyte recruitment [120]. Hence, CXCR4-directed uptake of ^68^Ga-pentixafor and its correlation with atherosclerotic plaque burden, calcification, magnitude of carotid stenosis and other CAD risk factors support the use of CXCR4 as a biomarker for further assessment of vulnerable atherosclerotic lesions. Nevertheless, data from CXCR4 imaging studies should be interpreted with caution, as CXCR4 expression is also beneficial in some cells, in contrast to its role in inflammatory cells [87].

Owing to the pivotal role of CXCR4 discussed above, CXCR4-targeted gene-transfer technology and molecular imaging-guided therapies are being tried to rectify atherosclerotic plaque progression and myocardial repair after myocardial infarction. PET-guided CXCR4 inhibition by AM3100 in CXCR4-upregulated cells demonstrated improved left ventricular outcomes in murine myocardial-infarction models [121]. In another report, CXCR4-targeted endoradiotherapy by ^90^Y-labeled Pentixather attenuated CXCR4 overexpression in atherosclerotic lesion areas, as confirmed by reduced ^68^Ga-pentixafor uptake. Although not confirmed by immunohistochemical analysis, CXCR4-targeted endoradiotherapy may open a new avenue for regional therapeutic targeting [122].

A phase II trial evaluated the role of POL6326 (a CXCR4 antagonist) as a stem-cell mobilizer and whether it can improve heart function after myocardial infarction (NCT01905475; EudraCT 2012- 003229-91). Another study reported that longevity-associated variant (LAV)-BPIFB4 gene therapy in a murine model of atherosclerosis exerts its therapeutic effect through modulation of CXCR4 because this therapeutic effect, which includes enhancing the favourable M2/M1 macrophage ratio, restoring endothelial function, decreasing T-cell proliferation and inhibiting the release of proinflammatory cytokines, was blunted by AMD3100 (a CXCR4 inhibitor) [123,97] reported that increased CXCR4 expression in human B-1 cells is associated with a distinct atheroprotective IgM repertoire and decreased coronary plaque burden; thus, therapeutic intervention to increase B-1 CXCR4 expression might be considered as a novel approach to limit atherosclerotic plaque progression [97].

From the above discussion, it is clear that therapeutic downregulation of CXCL12 or boosting of its receptors CXCR4/ACKR3 can attenuate inflammatory atherogenic plaque progression. However, a universal approach bears the considerable risk of adverse effects due to the functional heterogeneity of CXCR4 or CXCR4/ACKR3 in different cells involved in angiogenesis and repair. Thus, it is recommended to regionally enhance CXCR4 expression in vascular ECs (but not in bone marrow-derived/haematopoietic cells) through the application of nanoparticle-based or polymer-based specific modulators by perivascular/intra-arterial routes or other selective routes.

## Conclusion

Chemokines and their receptors play pivotal roles in the initiation, development and progression of atherosclerosis. The CXCL12/CXCR4/ACKR3 axis has been recently reported to perform more complex functions than previously thought; furthermore, these functions can be proatherogenic and atheroprotective, depending on cell type, downstream pathway and physiological state. Thus, CXCL12 and its receptors are speculated to be potential therapeutic targets and biomarkers in atherosclerosis and other CVDs. Specific temporal and spatial modulators of CXCL12, CXCR4, and ACKR3, in addition to specific modulators of downstream signalling, should be explored to suppress their unwanted action in the pathogenesis of atherosclerosis and to enhance their desirable effects. However, owing to complex ligand–receptor and receptor–receptor interactions in different cell types and heterogeneity in downstream signalling pathways, chemokine-based therapeutics require extensive experimentation, including studies employing fluorescent labelling, molecular imaging, genetic engineering and computational biology approaches.
